# The Prognostic Value of Olfactory Dysfunction in Patients with COVID-19: The COVIDORA Study

**DOI:** 10.3390/life14030293

**Published:** 2024-02-22

**Authors:** Anne-Laure Hamel, Léo Delbos, Pierre-André Natella, Thomas Radulesco, Mihaela Alexandru, Emmanuel Bartaire, Sophie Bartier, Gonda Benoite, Emilie Bequignon, Laurent Castillo, Florence Canouï-Poitrine, Florent Carsuzaa, Alain Corré, André Coste, Vincent Couloigner, Clémentine Daveau, Paul De Boissieu, Guillaume De Bonnecaze, Ludovic De Gabory, Christian Debry, Simon Deraedt, Xavier Dufour, Wissame El Bakkouri, Laurent Gilain, Stéphane Hans, Charlotte Hautefort, Ruben Hermann, Roger Jankowski, Candice La Croix, Jean-Baptiste Lecanu, Olivier Malard, Justin Michel, Yann Nguyen, Jerome Nevoux, Jean-François Papon, Vincent Patron, Marine Prigent, Virginie Pruliere-Escabasse, Marion Renaud, Cécile Rumeau, Dominique Salmon, Nicolas Saroul, Elie Serrano, Christine Nhung Tran Khai, Stéphane Tringali, Eric Truy, Clair Vandersteen, Benjamin Verillaud, Raphaël Veil, Maxime Fieux

**Affiliations:** 1 URC, Hôpital Henri Mondor, Assistance Publique des Hôpitaux de Paris, F-94010 Créteil, France; anne-laure.hamel@hotmail.fr (A.-L.H.); pierre.natella@aphp.fr (P.-A.N.); 2 Service de Santé Publique et d’Épidémiologie, Hôpital Bicêtre, Assistance Publique des Hôpitaux de Paris, F-94270 Paris, France; leodelbos31@gmail.com (L.D.); paul.de-boissieu@universite-paris-saclay.fr (P.D.B.); raphael.veil@gmail.com (R.V.); 3 Université Paris-Saclay, F-91190 Paris, France; mihaela.dana.alexandru@gmail.com (M.A.); jerome.nevoux@aphp.fr (J.N.); jean-francois.papon@aphp.fr (J.-F.P.);; 4 Department of Oto-Rhino-Laryngology Head and Neck Surgery, La Conception University Hospital, Aix-Marseille Univesity, F-13005 Marseille, France; thomas.radulesco@ap-hm.fr (T.R.); justin.michel@ap-hm.fr (J.M.); 5 Aix Marseille University, APHM, CNRS, IUSTI, F-13005 Marseille, France; 6 Assistance Publique-Hôpitaux de Paris (AP-HP), Hôpital Bicêtre, Service d’ORL, F-94270 Paris, France; 7 INSERM, UMR-S933 Genetic Diseases of Pediatric Expression, Sorbonne University, 75005 Paris, France; 8 GHICL, Hôpital Saint-Vincent-de-Paul, F-59000 Lille, France; bartaire@gmail.com; 9 Centre de Santé SOMeD, F-59777 Lille, France; 10 Centre de Santé Richerand, F-75010 Paris, France; 11 Service d’ORL et de Chirurgie Cervico Faciale, Hôpital Henri Mondor, Assistance Publique des Hôpitaux de Paris, F-94010 Créteil, France; sophiebartier@hotmail.fr; 12 Univ Paris Est Creteil, INSERM, IMRB, F-94010 Créteil, France; emilie.bequignon@gmail.com (E.B.); florence.canoui-poitrine@aphp.fr (F.C.-P.); andre.coste@chicreteil.fr (A.C.); virginie.escabasse@chicreteil.fr (V.P.-E.); 13 CNRS EMR 7000, F-94010 Créteil, France; 14 Délégation à la Recherche Clinique et à l’Innovation, GHICL, Hôpital Saint-Vincent-de-Paul, Faculté Libre de Médecine de Lille, F-59000 Lille, France; gonda.benoite@ghicl.net; 15 Service d’ORL et de Chirurgie Cervico Faciale, Centre Hospitalier Inter Communal de Créteil, F-94010 Créteil, France; 16 Institut Universitaire de la Face et du Cou, Centre Hospitalier Universitaire, Université Côte d’Azur, F-06100 Nice, France; castillo.l@chu-nice.fr (L.C.)); vandersteen.c@chu-nice.fr (C.V.); 17 Service de Santé Publique, Hôpital Henri Mondor, Assistance Publique des Hôpitaux de Paris, F-94010 Créteil, France; 18 Service d’ORL, de Chirurgie Cervico-Faciale et d’Audiophonologie, Centre Hospitalier Universitaire de Poitiers, F-86000 Poitiers, France; florent.carsuzaa@gmail.com (F.C.); xavier.dufour@chu-poitiers.fr (X.D.); 19 LITEC UR15560, Université de Poitiers, F-86000 Poitiers, France; 20 Service d’ORL et de Chirurgie Cervico Faciale, Hopital Fondation Rothschild, F-75019 Paris, France; al.corre75@free.fr (A.C.); welbakkouri@for.paris (W.E.B.); 21 Service d’ORL Pédiatrique, Hôpital Necker-Enfants Malades, Assistance Publique—Hôpitaux de Paris, F-75015 Paris, France; vincent.couloigner@aphp.fr; 22 Université Paris Cité, F-75015 Paris, France; 23 Service d’ORL et de Chirurgie Cervico Faciale, Hôpital de la Croix Rousse, Hospices Civils de Lyon, F-69004 Lyon, France; clementine.daveau@chu-lyon.fr; 24 Service d’ORL et Chirurgie Cervico-Faciale, Pôle Clinique des Voies Respiratoires, Hôpital Larrey, F-31059 Toulouse, France; debonnecaze.g@chu-toulouse.fr (G.D.B.); serrano.e@chu-toulouse.fr (E.S.); 25 Université Paul Sabatier Toulouse III, F-31062 Toulouse, France; 26 Université Paul Sabatier Toulouse III, Laboratoire Center for Anthropobiology and Genomics of Toulouse, F-31059 Toulouse, France; 27 Service d’ORL et Chirurgie Cervico-Faciale, Centre Hospitalo-Universitaire de Bordeaux, F-33000 Bordeaux, France; ludovic.de-gabory@chu-bordeaux.fr; 28 Service d’ORL et de Chirurgie Cervico-Faciale, Hôpitaux Universitaires de Strasbourg, Hôpital de Hautepierre, F-67200 Strasbourg, France; christian.debry@chru-strasbourg.fr (C.D.); renaud.marion@hotmail.fr (M.R.); 29 Unité INSERM 1121 Biomatériaux et Bioingénierie, CRBS, F-67000 Strasbourg, France; 30 Service d’ORL et de Chirurgie Cervico-Faciale, GHICL, Hôpital Saint-Vincent-de-Paul, Faculté Libre de Médecine de Lille, F-59000 Lille, France; deraedt.simon@ghicl.net; 31 Service d’ORL et de Chirurgie Cervico-Faciale, Hôpital Universitaire Gabriel Montpied, F-63000 Clermont-Ferrand, France; lfgilain@gmail.com (L.G.); nsaroul@chu-clermontferrand.fr (N.S.); 32 Unité de Nutrition Humaine, Équipe ASMS UMR 1019, Université Clermont Auvergne, INRAE, CNRH Auvergne, F-63000 Clermont-Ferrand, France; 33 Service d’ORL et de Chirurgie Cervico-Faciale, Hôpital Foch, Université Paris Saclay, F-92150 Paris, France; prhans.foch@gmail.com; 34 Service d’ORL et de Chirurgie Cervico Faciale, Hôpital Lariboisière, Assistance Publique des Hôpitaux de Paris, F-75010 Paris, France; charlotte.hautefort@aphp.fr (C.H.); benjamin.verillaud@aphp.fr (B.V.); 35 INSERM U1141, Université Paris Cité, Hôpital Robert Debré, F-75019 Paris, France; 36 Service d’ORL, de Chirurgie Cervico-Faciale et d’Audiophonologie, Hôpital Edouard Herriot, Hospices Civils de Lyon, F-69003 Lyon, France; ruben.hermann@chu-lyon.fr (R.H.); eric.truy@chu-lyon.fr (E.T.); 37 INSERM U1028, CNRS UMR5292, Lyon Neuroscience Research Center, Equipe IMPACT, F-69500 Bron, France; 38 Université de Lyon, Université Lyon 1, F-69003 Lyon, France; stephane.tringali@chu-lyon.fr; 39 Université de Lorraine, CHRU-Nancy, Service ORL, F-54000 Nancy, France; r.jankowski@chru-nancy.fr (R.J.); c.rumeau@chru-nancy.fr (C.R.); 40 Université de Lorraine, DevAH, F-54000 Nancy, France; 41 Service d’ORL et de Chirurgie Cervico Faciale, Hôpital Cochin, Assistance Publique des Hôpitaux de Paris, F-75014 Paris, France; candice.lacroix@aphp.fr; 42 Service d’ORL et de Chirurgie Cervico Faciale, Institut Arthur Vernes, F-75006 Paris, France; jblecanu@institut-vernes.fr; 43 Service d’ORL et de Chirurgie Cervico-Faciale, Hôpital Universitaire de Nantes, Centre Hospitalier Universitaire de Nante, 1 Place A. Ricordeau Hôtel-Dieu, F-44093 Nantes, France; olivier.malard@chu-nantes.fr; 44 INSERM, UMRS 1229, Regenerative Medicine and Skeleton (RMeS), F-44093 Nantes, France; 45 Service ORL, GHU Pitié-Salpêtrière, Assistance Publique-Hôpitaux de Paris (AP-HP), F-75013 Paris, France; nguyenyann@hotmail.com; 46 Sorbonne Université, Faculté de Médecine, F-75013 Paris, France; 47 Service d’ORL et de Chirurgie Cervico Faciale, Hôpital Universitaire de Caen, F-14000 Caen, France; patron-v@chu-caen.fr; 48 Service d’ORL et de Chirurgie Cervico Faciale, Hôpital Robert Debré, Assistance Publique des Hôpitaux de Paris, F-75019 Paris, France; marinepri@hotmail.com; 49 Assistance Publique Hôpitaux de Paris, Direction des relations internationales, Université Paris Cité, F-75270 Paris, France; dominique.salmon@aphp.fr; 50 Hospices Civils de Lyon, Centre Hospitalier Lyon Sud, Service d’ORL, d’Otoneurochirurgie et de Chirurgie Cervico-Faciale, F-69310 Pierre Bénite, France; 51 UMR 5305, Laboratoire de Biologie Tissulaire et d’Ingénierie Thérapeutique, Institut de Biologie et Chimie des Protéines, CNRS/Université Claude Bernard Lyon 1, F-69367 Lyon, France; 52 Laboratoire CoBTeK, Université Côte d’Azur, F-06100 Nice, France

**Keywords:** olfactory dysfunction, anosmia, phantosmia, parosmia, COVID-19, taste dysfunction, risk factors, diabetes, hypertension, renal insufficiency, tobacco

## Abstract

Background: Among all studies describing COVID-19 clinical features during the first wave of the pandemic, only a few retrospective studies have assessed the correlation between olfac-tory dysfunction (OD) and the evolution of disease severity. The main aim was to assess whether OD is a predictive factor of COVID-19 severity based on the patient’s medical management (outpa-tient care, standard hospital admission, and ICU admission). Methods: A national, prospective, mul-ticenter cohort study was conducted in 20 public hospitals and a public center for COVID-19 screen-ing. During the first wave of the pandemic, from 6 April to 11 May 2020, all patients tested positive for COVID-19 confirmed by RT-PCR underwent two follow-up ENT consultations within 10 days of symptom onset. The main outcome measures were the evolution of medical management (out-patient care, standard hospital admission, and ICU admission) at diagnosis and along the clinical course of COVID-19 disease. Results: Among 481 patients included, the prevalence of OD was 60.7%, and it affected mostly female patients (74.3%) under 65 years old (92.5%), with fewer comor-bidities than patients with normal olfactory function. Here, 99.3% (290/292) of patients with OD presented with non-severe COVID-19 disease. Patients reporting OD were significantly less hospi-talized than the ones managed as outpatients, in either a standard medical unit or an ICU. Conclu-sions: As regards the clinical course of COVID-19 disease, OD could predict a decreased risk of hospitalization during the first wave of the pandemic.

## 1. Introduction

Three years after the first description of coronavirus disease 19 (COVID-19) in the Chinese town of Wuhan, and as the world is still at risk of new waves of the pandemic, olfactory and taste dysfunction (OTD) induced by the infection is still subject to unresolved issues. Whereas the main clinical features found in the literature in the early months of the outbreak appeared to be less specific [1,2], with fever, dry cough, upper-airway infections, and fatigue at the forefront [3], some European studies started to notice a surge of chemosensory dysfunction without concomitant nasopharyngeal symptoms [4,5] around March 2020. Ever since, several research teams around the globe have investigated OTD in COVID-19 patients, no matter the difficulties encountered [6,7,8,9]. The assessment of chemosensory dysfunctions, specifically the non-significant correlation between subjective symptoms and semi-objective chemosensory testing, makes the assessment of OD symptoms related to COVID-19 difficult [10]. The authors provided insight into epidemiologic characteristics, suggesting that OTD concerned a significant number of patients with a mild to moderate form of the disease [11]. However, most studies assessing such an association between olfactory dysfunction (OD) and the clinical course of COVID-19 were retrospective, conducted in a single center, and had a cross-sectional design [12]. Nevertheless, some advantages could be mentioned when authors are using a retrospective design: a long-term follow-up becomes possible, and persistent OD related to COVID-19 can be investigated.

Thus, to assess the association between OD and COVID-19 severity and then to clearly assess its prognostic value, we decided to conduct a national multicenter prospective study. A cohort of patients across France with a severe acute respiratory syndrome coronavirus 2 (SARS-CoV-2) infection confirmed by real-time polymerase chain reaction (RT-PCR)) [13], all clinical presentations combined, were included.

The main objective was to assess whether OD could be a predictive factor of COVID-19 disease severity based on the patient’s required medical management (outpatient care vs. standard hospital admission, and outpatient care vs. Intensive Care Unit (ICU) admission) within ten days following a positive RT-PCR test. Secondary objectives were to identify other risk factors leading to hospitalization (standard or ICU admission) and to assess the prevalence of OD in a population of COVID-19-positive patients, as well as to characterize this specific population.

## 2. Materials and Methods

### 2.1. Study Design

This nation-wide, prospective, multicenter cohort study was conducted from 6 April to 11 May 2020, by ear, nose, and throat (ENT) physicians in 20 public hospitals across France and by an infectious disease specialist at the Paris screening center for COVID-19 (21 medical centers in total). In each one, on the basis of all the SARS-CoV-2 RT-PCR tests carried out on one day, consecutive patients were included if they tested positive for COVID-19 by SARS-CoV-2 RT-PCR assay using a respiratory sample (nasopharyngeal swabs or induced sputum specimen). Patients with other risk factors for OD other than a SARS-CoV-2 infection were excluded. Indeed, exclusion criteria were medical history associated with a risk of OD (chronic rhinosinusitis (with or without nasal polyps in the nasal fossa), previous nasal operations (e.g., FESS or rhinoplasty), inflammatory or neurodegenerative neurological pathology, and current or previous chemotherapy or cerebral radiation therapy) and known OD of any cause other than SARS-CoV-2 infection (including those with a history of head trauma). Patient follow-up consisted of two consecutive consultations, respectively, within 3 days and 7–10 days following inclusion, either through a phone call or a face-to-face consultation. The consultation process followed a Case Report Form (CRF)-based questionnaire designed by the authors. For patients who were in no condition to respond on the assumed dates of the follow-up consultations (for instance, if they were in the ICU), data collection was performed upon improvement of the patient’s condition.

### 2.2. Ethics

The study was conducted in accordance with the Declaration of Helsinki and was approved by the local Institutional Review Board of Henri-Mondor Hospital (Ethics Committee number: 00011558; Approval number: 2020_058). All data were anonymized, and all patients gave informed consent.

### 2.3. Population

During the first consultation (C1), which occurred within three days after the SARS-CoV-2 positive RT-PCR result, the patients were asked to answer specific questions regarding demographic data (age, gender, weight, height, occupation, and tobacco use), their medical history (allergy, chronic disease, immunosuppression, diabetes, and chronic hypertension), history of rhinological conditions (chronic rhinosinusitis and history of OD), and usual personal treatments. OD clinical features such as date, onset (sudden or progressive), and severity of associated olfactory symptoms (parosmia and phantosmia) as well as taste dysfunction (TD) were also collected with nose-related symptoms (nasal discharge and obstruction, sneezing, nasal pain, and paresthesia). We recorded the duration and type of treatment for OD (nasal irrigation with saline or budesonide, topical and/or systemic corticosteroid treatment, antibiotics, omega-3 diet, or topical vitamin A). The data specifically related to COVID-19 included clinical history with common symptoms of the disease and the initial type of medical management provided, such as outpatient care, standard hospital admission, or admission in an ICU.

We conducted the second consultation (C2) within seven to ten days after the SARS-CoV-2 RT-PCR result and collected the following data: evolution of OD and rhinological symptoms (nasal discharge and obstruction, sneezing, nasal pain, and paresthesia) and other signs of COVID-19, evolution in the patient’s management, such as hospitalization in a medical unit or an ICU. To assess the clinical severity of COVID-19 reached by each patient during the disease, we constructed a “worst clinical situation” variable corresponding to the maximum level of care required (ICU admission > standard hospital admission > outpatient care) based on C1 and C2 reported medical management.

### 2.4. Statistical Analysis 

To assess whether OD could be a predictive factor of COVID-19 disease severity based on the patient’s medical management during the ten days following a positive RT-PCR test and to identify other risk factors, univariate analyses using logistic regressions were performed, testing standard hospital admission (severe) against outpatient care (non-severe) on the one hand and admission in an ICU (very severe) against outpatient care (non-severe) on the other hand. Because of the relatively low number of ICU-admitted patients in our sample (n = 20 considering the medical management corresponding to the “worst clinical situation”), a limited number of independent variables in our analyses were included. Besides the variable of interest (OD), we selected variables previously described in the literature as risk factors for hospitalization and severe outcomes in COVID-19 disease, such as age, male sex, obesity, hypertension, and diabetes [14,15,16]. We included these five variables in multinomial regression models to perform multivariate analyses. Results for all regressions were reported as odds ratios (OR) with their corresponding confidence intervals at 95% (95% CI). The patient’s age was a potential confounding covariate in two distinct ways: older age increased the risk of a severe form of the disease, yet it could also increase the risk of being denied admission to an ICU (older patients often meet criteria for non-ICU admission), which we use as a proxy to measure disease severity. Since no patient in an ICU was over 80 years old (maximum: 79 years old), we excluded patients over 80 years of age from the analysis (n = 14). 

To assess the prevalence of OD and to characterize the population, clinical features of COVID-19 at C1 using n (%) were obtained and then tested to identify whether there was a significant difference between inpatients (admitted either in a standard medical unit or in an ICU) and outpatients according to the “worst clinical situation”. 

To assess the sample size regarding the primary objective, we considered a minimal requirement of 10 events per parameter in our model. The expected distribution of the clinical severity spectrum of COVID-19 was described by Wu and McGoogan in the early phase of the pandemic as approximately 20% of patients with a moderate-to-severe form of the disease [17]. To build a robust multivariate model with six parameters, one main explanatory variable, and the five adjustment covariates chosen (age, sex, obesity, diabetes, and hypertension), we needed a minimum of 60 events and thus a minimal sample size of 300 patients. Regarding our secondary objective, the expected prevalence of OD was 53% in a meta-analysis with more than 1200 patients published early in 2020 [9], and we aimed for a 5% precision with a 95% confidence interval, leading to a minimum sample size of 383 patients. Considering a 20% rate of missing data and/or loss of follow-up, we intended to include at least 479 patients. Baseline demographic and medical characteristics of the patients were described by n (%) and compared between patients with and without OD at the first consultation using a Chi-squared test (or Fisher’s exact test when necessary) for non-ordinal categorical covariates and the Cochran-Armitage test for ordinal covariates.

The statistical analyses were performed using R software (v. 1.3.10703, R Foundation for Statistical Computing, Vienna, Austria, www.r-project.org (accessed on 1 September 2020)). *p*-values were calculated using two-tailed tests. 

## 3. Results

### 3.1. Baseline Demographics and Evolution of Clinical Courses along Follow-Up

**Figure 1:**
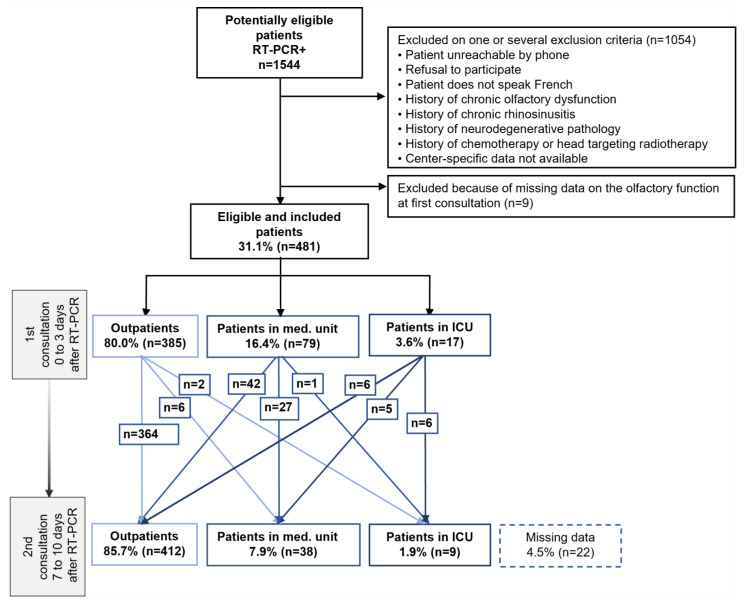
Flow chart of an observational, prospective multicenter cohort study of patients screened positive for COVID-19 by a positive SARS-CoV-2 RT-PCR. Footnotes: A follow-up with two consultations within 7 to 10 days of the patient’s symptoms and his management in relation to the natural course of the disease. Abbreviations: ICU, intensive care unit; Med. Unit, medicine unit; RT-PCR, reverse transcriptase polymerase chain reaction.

Of the 1544 consecutive eligible patients with SARS-CoV-2-positive RT-PCR, 481 patients were included (details in Figure 1). Among the 481 patients included, we recorded at the first consultation: 80% of outpatients (n = 385/481), 16.4% of patients admitted to a medical unit (n = 79/481), and 3.5% of patients admitted to an ICU (n = 17/481). At the second consultation, seven to ten days later, there were 85.7% of outpatients (n = 412/481), 7.9% of patients in a medical unit (n = 38/481), and 1.9% of patients in an ICU (n = 9/481); data were missing for 22 patients. Among the patients who were discharged after their RT-PCR at the first consultation, only 2.1% (n = 8/385) were hospitalized between the first and second consultations; 1.6% of them went into a medical unit (n = 6/385) and 0.5% into an ICU (n = 2/385); data were missing for 13 patients. One patient who was hospitalized in a medical unit at the time of the first consultation was transferred to an ICU by the time of the second consultation. Overall, there was a 1.9% (n = 9/481) unfavorable trend in medical management in our total population between the first and second consultations, whereas 11% (n = 53/481) of patients experienced improvement in their clinical situation during follow-up. Details appear in the flow chart of the study (Figure 1).

Demographics and comorbidities are shown in Table 1, according to the status of olfactory function at the first consultation. Overall, patients were 45.4 years old, female in 67.8% (n = 326/481) of cases, non-smokers (67.4%, n = 324/481), and mostly working as healthcare personnel (57.2%, n = 275/481). 

**Figure 2:**
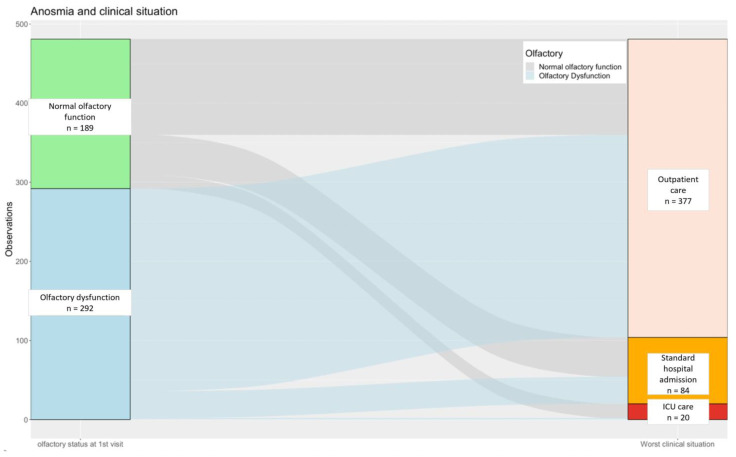
Alluvial plot of the distribution of patients’ “worst clinical situation” along follow-up according to olfactory function status at first consultation. Footnotes: We used the proxy of the “worst clinical situation” for each patient corresponding to the heavier medical care needed along the course of the disease between C1 and C2. Numbers (n) cor-respond to patients. Abbreviations: ICU, intensive care unit.

Using the proxy of the “worst clinical situation” throughout follow-up depending on OD at first consultation, we recorded 87.7% of outpatients (n = 256/481), 11.6% of patients hospitalized in a medical unit (n = 34/481), and 0.7% of patients admitted to an ICU (n = 2/481). We used an alluvial plot to illustrate this distribution of disease severity throughout follow-up (Figure 2).

### 3.2. Predictive Factors Associated with Hospitalization

Univariate and multivariate analysis results are presented in Table 2. Independent risk factors for hospitalization in a medical unit or in an ICU were male gender (adjusted aOR 2.62 [1.49–4.63], *p* < 0.001 and aOR 5.97 [1.71–20.8] *p* = 0.005, respectively) and age above 65 years old (aOR 6.31 [2.86–13.96] *p* < 0.001 and aOR 5.02 [1.34–18.8] *p* = 0.017, respectively). However, obesity (BMI > 30), diabetes, and hypertension were not significantly associated with hospitalization in a medical unit when adjusted for other covariates. Diabetes was an independent risk factor for hospitalization in the ICU (aOR 11.59 [3.36, 40.0] *p* < 0.001), but not in a medical unit (1.95 [0.80, 4.79] *p* = 0.14). OD was found to be inversely associated with hospitalization when compared to outpatient management in a medical unit (OR 0.51 [0.29–0.89] *p* = 0.018) and in an ICU (OR 0.09 [0.02–0.43] *p* = 0.003); it is a protective factor for hospitalization. 

### 3.3. Prevalence of OD

At the first consultation, the prevalence of OD was 60.7% (n = 292/481, patients reporting anosmia or hyposmia, and no missing data), and the prevalence of TD was 54% (n = 260/481, missing data for 2 patients). Patients with OD were significantly younger than those in the group with self-reported normal olfactory function (respectively, 92.5% (n = 270/292) and 77.8% (n = 147/189) under 65 years old, *p* < 0.001). Female patients were significantly more frequent in the OD group (74.3% (n = 217/292)) than in the group with normal olfactory function (57.7% (n = 109/189), *p* < 0.001). Patients in the OD group also had significantly fewer comorbidities, especially diabetes (6.5%, n = 19/292, *p* = 0.002), arterial hypertension (13%, n = 38/292, *p* < 0.001), and renal insufficiency (1.4%, n = 4/292, *p* < 0.001). Details are shown in Table 1. 

Among the 292 patients presenting with OD at the first consultation, the onset of the disorder was isolated for 18.8% (n = 55/292, missing data for n = 3) and was mostly sudden (66.1%, n = 193/292, missing data for n = 2). Regarding the severity of OD, 161 patients (55.1%, n = 161/292) reported complete loss of smell, whereas 124 (42.5%, n = 124/292) patients reported only a partial OD; data were missing for 7 patients. Parosmia and phantosmia were reported in, respectively, 9.6% (n = 28/292, no missing data) and 7.9% of cases (n = 23/292, missing data for n = 1). TD reported at the first consultation occurred as an abnormal perception of flavors for 90.0% (n = 234/260, missing data for n = 9) of patients presenting with TD, but also an abnormal perception of taste, such as for salty, sweet, bitter, and acidic foods, for 62.0% (n = 161/260, missing data for n = 15) of patients presenting with TD. Moreover, 54.7% (n = 142/260, missing data for n = 17) of patients presented both characteristics of TD.

### 3.4. Clinical Features of COVID-19 Disease

The clinical features of COVID-19 at the first consultation according to whether patients were hospitalized or not during follow-up are also shown in Table 3. Hospitalized patients presented with fever (78.8% (n = 82/104) *p* < 0.001), cough (74.0% (n = 77/104) *p* = 0.023), and dyspnea (62.5% (n = 65/104) *p* < 0.001) significantly more often than outpatients (52.8% (n = 199/377), 61.8% (n = 233/377), and 24.9% (n = 94/377), respectively, *p* < 0.001, *p* = 0.023, and *p* < 0.001), and significantly fewer of them reported myalgia (40.4%, n = 42/104) and headaches (49.0%, n = 51/104) than outpatients (62.1% (n = 234/377) and 72.4% (n = 273/377), respectively, *p* < 0.001 and *p* < 0.001). Asthenia was a symptom common to most patients in both groups (89.4% of inpatients (n = 93/104) and 82.0% (n = 309/377) of outpatients, *p* = 0.076). Nasal symptoms like rhinorrhea and nasal obstruction were reported significantly more by outpatients than inpatients (42.2% (n = 159/377) vs. 18.3% (n = 19/104), *p* < 0.001, and 35.0% (n = 132/377) vs. 20.2% (n = 21/104), *p* = 0.013, respectively). Considering the presence of chemosensory dysfunction, OD and TD were reported significantly more by outpatients than inpatients, respectively, 67.9% (n = 256/377) and 60.2% (n = 227/377) vs. 34.6% (n = 36/104) and 31.7% (n = 33/104) (*p* < 0.001 and *p* < 0.001). Moreover, simultaneous OD and TD were more frequent in outpatients (57.3%, n = 216/377) than inpatients (24.0%, n = 25/104). 

## 4. Discussion

These results suggest that OD in COVID-19 patients infected during the first wave of the pandemic was a protective factor for hospitalization in both the medical unit and the ICU after adjusting for potential confounding factors. The prevalence of OD after COVID-19 diagnosis was 60.7% (292/481), and patients were mostly female under 65 years old. These findings suggest, consistent with the literature, that OD as an early symptom of COVID-19 during the first wave of the pandemic could predict a mild to moderate form of the disease not requiring hospitalization [11,12]. The aim of our study was indeed to study the predictive nature of OD in relation to the level of medical management required along the course of COVID-19 and not to provide a causal explanation for the potential severity of the disease.

In line with the literature, we found that age above 65 years, male gender, and frequent comorbidities, such as arterial hypertension, were risk factors associated either with hospitalization in a medical unit or in an ICU during the clinical course of the SARS-CoV-2 infection [18,19]. In line with some study results [20,21], diabetes was not found to be an independent risk factor for hospitalization in a medical unit but for hospitalization in the ICU. This could be explained by their common association with other cardiovascular risk factors and the complications they can generate [21]. Surprisingly, obesity, though frequently found in patients hospitalized with COVID-19 [22], was not identified as a risk factor for hospitalization. 

60.7% (n = 292/481) of patients reported OD in our population of patients who tested positive for SARS-CoV-2, which is consistent with the literature on OD within Western countries during the first wave of the pandemic [8,9,23,24], including a meta-analysis on 27,492 patients who reported an OD prevalence of 54% for European studies in their subgroup analyses by continent [24]. Even though the prevalence of smell impairment has decreased all over the world with the spread of new variants of SARS-CoV-2 [25,26,27], how can we explain such a high prevalence of this symptom during the early phase of the pandemic in Western countries [24]. If some parts of the pathophysiology of OD in COVID-19 disease remain unclear, the main underlying mechanisms are now well known. First, it is established that infection with the SARS-CoV-2 virus involves interactions between its spike (S) protein and angiotensin-converting enzyme II (ACE2) on target cells, with this interaction requiring cleavage of the S protein by the cell surface protease called transmembrane protease serine 2 (TMPRSS2) [28,29]. Thus, cells with high ACE2 and TMPRSS2 expression have strong virus-binding ability and are particularly prone to infection [30]. A part of the explanation could lie in the hypothesis of a higher affinity of the virus for the ACE2 receptor than other viruses usually find in common colds, like other coronaviruses; indeed, the study of Wrapp et al. highlighted that this affinity was ten times higher for SARS-CoV-2 than SARS-CoV-1 [31]. As it happens, those two proteins are abundantly expressed by supportive cells of the olfactory epithelium called sustentacular cells [32,33], and it is now admitted that the destruction of these cells resulting from their invasion by the virus may temporarily damage the epithelium [34] and thus mediate OD [35]. Regarding the decline of OD prevalence with the Omicron variant, two main hypotheses have been made and could be complementary. First, the study of Omicron’s genomic sequence found mutations in the spike protein, which tend to make the virus more hydrophobic [36], interfering with its solubility within the mucus [35]. Second, some other mutations may cause less efficient cleavage by TMPRSS2, resulting in reduced surface membrane fusion mediated by this protein [34,35,36]. This could ultimately lead Omicron to use the endosomal route as another pathway to enter the cell, making it less efficient in infecting the sustentacular cells [37,38]. These findings could also explain the recovery rate from chemosensory dysfunctions reported with the Omicron BA.1 subvariant, which was more favorable with a shorter duration [39]. It could also have been positively influenced by vaccination [39].

Regarding the great disparity in OD prevalence among world regions during the early time of the pandemic, Shelton et al. have suggested that it could be related to a host factor involving genetics, with a locus named UGT2A1/A2 encoding the uridine 5’-diphospho (UDP) glycosyltransferase, an enzyme related to olfaction [40]. This protein, highly conserved between species [41,42], is thought to play several roles within olfactory perception, from terminating odorant signal transduction [43] to preventing saturation of the odorant receptors [41]. Shelton et al.’s genome-wide association study highlighted different profiles regarding the UGT2A1/A2 risk allele frequency between ethnic groups around the world; these differences significantly match the discrepancies in OD prevalence with the same pattern [40]. Thus, the greater degree of OD caused by the risk allele at the UGT2A1/A2 locus may explain the greatest susceptibility to loss of smell in Western countries [40], in addition to polymorphisms affecting this enzyme that could play a part in inter-individual variability in olfactory sensitivity [42]. Despite the decline of OD prevalence around the world due to the rise of the Omicron variant, von Bartheld and Wang meta-analysis revealed the same pattern of significant ethnic differences regarding omicron-induced OD prevalence in adults, estimated at 11.7% in Western countries compared to 3.7% globally [28].

As previously described [24,44], patients reporting OD in our study were generally middle-aged, mostly female. This finding could also be partly explained by the difference in UDP glycosyltransferase expression with aging on the one hand [45] and between genders on the other hand (female-predominant expression) [46]. And another explanation could lie in the physiological age and sex differences in the immune response to SARS-CoV-2 infection. Some studies have highlighted the variability in immune response between adults with a moderate-to-severe form of the disease and pediatric patients; SARS-CoV-2 infection leads to increased production of inflammatory markers in both adults and children, associated with a decreased number of circulating CD4+ and CD8+ lymphocytes in adults, especially with a severe clinical course [47,48]. There are also consistent differences in the immune reaction to COVID-19 in men and women; females seem better equipped to combat the viral infection, with immune features like T cell activity enhancement [49], decreased expression of ACE2 receptors in the lung, and inhibition of pro-inflammatory cytokine activity driven by female hormones like estrogen [49,50]. In light of these considerations, as suggested by Gori et al. [47] and Lechien et al. [51,52], the encounter between the SARS-CoV-2 virus and the olfactory epithelium could have been a key point in the clinical course of the disease, depending on patient characteristics. The development of a local immunological reaction could have restricted the infection to the olfactory epithelium, leading to a mild form of the disease with transient OD, as in the case of young female patients. Conversely, in other cases, uncontrolled viral replication in the nasal epithelium and the resulting spread of severe inflammation could have been responsible for more severe manifestations of COVID-19. The new key finding of our study, based on the COVID-19-positive population, is that the onset of OD could be linked to a mechanism protecting against the development of a severe form of COVID-19 disease and hospitalization, since 87.7% (n = 256/292) of patients with OD were eventually discharged and 11.6% (n = 34/292) were hospitalized in a medical unit, with protective adjusted OR in favor of less hospitalization and less ICU admission. Although a different gender distribution was described in this study, previous studies did not observe a gender difference considering chemosensory testing results (i.e., the severity of OD) [10]. Our findings regarding the OD phenotype are consistent with those of other authors, where the persistent smell and taste disorder phenotypes were characterized by good clinical, physical, and mental recovery as compared with convalescents affected by prolonged fatigue or neurocognitive complaints [53].

There was a very low rate of unfavorable trends in medical management in our total population between the first and second consultations, with only 1.9% (n = 9/481) of patients experiencing worsening clinical outcomes and requiring more intensive medical care before the second consultation, regardless of olfactory function at the first consultation. This is at odds with the natural course of the disease described by many studies over the past two years of the pandemic, in which the risk of clinical worsening during the first seven days of infection is related to a cytokine storm [54,55].

To our knowledge, our study is the only real-life prospective, multicenter study that has established through longitudinal follow-up the prognostic character of OD in COVID-19 disease during the first wave of the pandemic. Indeed, although numerous studies have been carried out on this subject, their designs were most often cross-sectional or retrospective [56,57]. Some studies used the same statistical analysis model to evaluate the association between OD and disease severity, but the outcome used was mortality [58]. Even though the prevalence of OD decreased with the appearance of the Omicron variant [59], these results are still of importance to predict disease severity, especially as we cannot predict the appearance of new SARS-CoV strains and the effects they will have on olfaction in the years to come. Moreover, we endeavored to include insights about OD among patients with severe disease requiring hospitalization in an ICU, an aspect of this patient population that has been scarcely studied. During the first wave of the COVID-19 pandemic, RT-PCR was only performed in hospitals, whereas this population included hospitalized patients and outpatients tested in a screening center. According to this method, we assumed obtaining a representative sample of the national population treated in inpatient and outpatient departments at this time of the pandemic.

This study, nonetheless, has several limitations. First, the comparison of the three groups of patients may be affected by some distinct bias inherent to their constitution. On the one hand, outpatients were interviewed more frequently during the first days of their symptoms and may have been more inclined to describe them precisely. Accordingly, in outpatient settings, notably more patients were observed, and even if it was clinically a correct distribution, it could have resulted in biases. On the other hand, data from hospitalized patients may have suffered from recall bias due to their general conditions and context of hospitalization, especially for those unable to answer questions at the time. This last characteristic concerned mostly the pool of ICU patients, for which the small sample size constituted a second limitation in this study. Furthermore, we excluded the patients over 80 years old (n = 14) from our models to limit misclassification bias since the clinical situation (outpatient, medical hospitalization, or ICU admission) was our proxy for severity but could also be inversely corrected to severity and general state regarding ICU admission (as some older patients may more often meet criteria for non-ICU admission). Third, we did not use specific validated olfactory tests or electrophysiological methods due to the emergency context at the time of data collection and the great spread of COVID-19. Nonetheless, several meta-analyses have revealed that most studies at the time of the pandemic involved online questionnaires only and that the prevalence of OD was higher with objective sensory testing than with subjective methods, with a prevalence of 72–77% vs. 44%, respectively [24,60]. As it turns out in our study, the prevalence of OD seems closer to the results obtained with objective tests. This finding may in part be explained by the specific features of the questionnaire (Supplementary Materials), which was designed by ENT physicians and conducted at least verbally, if not in person, rather than by an online auto-questionnaire. Lastly, the follow-up with our patients was too short to properly assess olfactory recovery.

## 5. Conclusions

This prospective, national real-life study was conducted during the initial wave of the COVID-19 pandemic in France. Our findings suggest a positive association between OD and a mild to moderate course of the disease, specifically with the first French variants. Consequently, OD was predictive of outpatient management during this period. Patients affected by OD were mostly young females without comorbidities, and even though the prevalence of this symptom is nowadays less common with the actual lull in the pandemic and the rising of Omicron variants, we humans are not yet sheltered from new strains of virus that could once again have a major impact on olfaction.

## Figures and Tables

**Table 1:** Population’s characteristics according to the olfactory status at first consultation (OD vs. normal olfactory function).

	Total: n = 481	OD at First Consultation: n = 292 (60.7%)	Normal Olfactory Function: n = 189 (39.3%)	*p*-Value
Sex female	326 (67.8%)	217 (74.3%)	109 (57.7%)	<0.001 *
Age				<0.001 *
<65 years	417 (86.7%)	270 (92.5%)	147 (77.8%)	
≥65 years	49 (10.2%)	19 (6.5%)	30 (15.9%)	
Body Mass Index (BMI)				0.62
<30	397 (82.5%)	243 (83.2%)	154 (81.5%)	
Obese	64 (13.3%)	49 (16.8%)	35 (18.5%)	
Tobacco				
Non-smoker	324 (67.4%)	189 (64.7%)	135 (71.4%)	0.027 *
History of tobacco use	142 (29.5%)	92 (31.5%)	50 (26.4%)	0.19
Comorbidities				
Immunosuppression	17 (3.5%)	8 (2.7%)	9 (4.8%)	0.247
Diabetes	48 (10%)	19 (6.5%)	29 (15.3%)	0.002 *
Hypertension	86 (17.9%)	38 (13%)	48 (25.4%)	<0.001 *
Renal insufficiency	20 (4.2%)	4 (1.4%)	16 (8.5%)	<0.001 *
Cancer	19 (4%)	11 (3.8%)	8 (4.2%)	0.81
Auto-immune disease	19 (4%)	12 (4.1%)	7 (3.7%)	0.83
Allergic rhinitis	120 (24.9%)	82 (28.1%)	38 (20.1%)	0.057
Personal treatments				
Antihypertensive drugs	78 (16.2%)	32 (11%)	46 (24.3%)	<0.001 *
Corticosteroids	11 (2.3%)	3 (1%)	8 (4.2%)	0.029 *
Non-steroidal anti-inflammatory drugs	2 (0.4%)	1 (0.3%)	1 (0.5%)	0.95
Antihistamine	21 (4.4%)	13 (4.5%)	8 (4.2%)	0.91
Work				<0.001 *
Liberal	90 (18.7%)	49 (16.8%)	41 (21.7%)	
Retirement	56 (11.6%)	17 (5.8%)	39 (20.6%)	
Unemployed	16 (3.3%)	7 (2.4%)	9 (4.8%)	
Healthcare worker	275 (57.2%)	195 (66.8%)	80 (42.3%)	
Other	40 (8.3%)	24 (8.2%)	16 (8.4%)	
Worst clinical situation				<0.001 *
Outpatient care	377 (78.4%)	256 (87.7%)	121 (64.0%)	
Hospitalized in medicine	84 (17.5%)	34 (11.6%)	50 (26.4%)	
Hospitalized in the ICU	20 (4.1%)	2 (0.7%)	18 (9.5%)	
Total hospitalization	104 (21.6%)	36 (12.3%)	68 (36.0%)	<0.001 *

Footnotes: Baseline demographic and medical characteristics of the patients were described by n (%) and compared between patients with and without OD at the first consultation using a Chi-squared test (or Fisher’s exact test when necessary) for non-ordinal categorical covariates, and the Cochran-Armitage test for ordinal covariates. * Indicates statistical significance (*p* < 0.05). Abbreviations: BMI, body mass index; OD, olfactory dysfunction; ICU, intensive care unit.

**Table 2:** Associated factors with hospitalization in medicine or in the intensive care unit compared to outpatient management.

	Standard Hospital Admission/Outpatient Care				Hospitalization in the ICU/Outpatient Care			
	Univariate Analyses		Multivariate Analyses		Univariate Analyses		Multivariate Analyses	
**Characteristics**	OR (95% CI)	*p*-value	**OR (95% CI)**	***p*-value**	OR (95% CI)	*p*-value	**OR (95% CI)**	***p*-value**
**OD at 1st cs**	0.32 [0.20, 0.52]	<0.001 *	**0.51 [0.29, 0.89]**	0.018 *	0.05 [0.01, 0.23]	<0.001 *	**0.09 [0.02, 0.43]**	0.003 *
**Age**								
**<65 years**	Ref		Ref		Ref		Ref	
**≥65 years**	10.1 [5.03, 20.2]	<0.001 *	**6.31 [2.86, 13.96]**	<0.001 *	17.2 [6.30, 47.1]	<0.001 *	**5.02 [1.34, 18.8]**	0.017 *
**Sex**								
**F**	Ref		Ref		Ref		Ref	
**M**	3.03 [1.86, 4.92]	<0.001 *	**2.62 [1.49, 4.63]**	<0.001 *	8.66 [3.07, 24.4]	<0.001 *	**5.97 [1.71, 20.8]**	0.005 *
**BMI in class**								
**BMI < 30**	Ref		Ref		Ref		Ref	
**Obese**	1.13 [0.62, 2.07]	0.69	**1.51 [0.76, 3.02]**	**0.24**	0.85 [0.24, 2.97]	0.80	**0.73 [0.16, 3.38]**	**0.69**
**Diabetes**								
**No**	Ref		Ref		Ref		Ref	
**Yes**	3.23 [1.57, 6.61]	<0.001 *	**1.95 [0.80,4.79]**	**0.14**	24.20 [8.97, 65.3]	<0.001 *	**11.59 [3.36, 40.0]**	<0.001 *
**Hypertension**								
**No**	Ref		Ref		Ref		Ref	
**Yes**	3.99 [2.31, 6.91]	<0.001 *	**1.26 [0.59, 2.71]**	**0.55**	14.1 [5.32, 37.1]	<0.001 *	**3.37 [0.92, 12.3]**	**0.067**

Footnotes: Univariate and multivariate analyses relied on multinomial logistic regressions. Results are provided in OR (95% CI). Variables and their adjusted OR are highlighted in bold. * Indicates statistical significance for adjusted OR (*p* < 0.05). Abbreviations: BMI, body mass index; OD, olfactory dysfunction; ICU, inten-sive care unit; Ref, reference value.

**Table 3:** Clinical features of COVID-19 at first consultation according to the worst clinical situation.

	Total (n = 481)	Outpatients (n = 377)	Inpatients (n = 104)	*p*-Value
Fever	281 (58.4%)	199 (52.8%)	82 (78.8%)	<0.001 ^a,^*
NA	4	4	0	
Cough	310 (64.4%)	233 (61.8%)	77 (74.0%)	0.023 ^a^
NA	1	1	0	
Dyspnea	159 (33.1%)	94 (24.9%)	65 (62.5%)	<0.001 ^a,^*
NA	1	1	0	
Asthenia	402 (83.6%)	309 (82.0%)	93 (89.4%)	0.076 ^a^
NA	1	1	0	
Myalgia	276 (57.4%)	234 (62.1%)	42 (40.4%)	<0.001 ^a,^*
NA	1	1	0	
Headache	324 (67.4%)	273 (72.4%)	51 (49.0%)	<0.001 ^a,^*
NA	2	1	1	
Rhinorrhea	178 (37.0%)	159 (42.2%)	19 (18.3%)	<0.001 ^b,^*
NA	6	3	3	
Nasal obstruction	153 (31.9%)	132 (35.0%)	21 (20.2%)	0.013 ^b,^*
NA	27	15	12	
Olfactory dysfunction	292 (60.7%)	256 (67.9%)	36 (34.6%)	<0.001 ^a,^*
NA	0	0	0	
Taste dysfunction	260 (54.1%)	227 (60.2%)	33 (31.7%)	<0.001 ^a,^*
NA	2	1	1	
Simultaneous OD and TD	241 (50.1%)	216 (57.3%)	25 (24.0%)	<0.001 ^b,^*
NA	0	0	0	

Footnotes: We used the proxy of the “worst clinical situation” for each patient, corresponding to the heavier medical care needed along the course of the disease between C1 and C2. Data are in n (%). Chi-squared test ^a^ (or Fisher’s exact test ^b^) was used for categorical covariates between outpatients and inpatients. * Indicates statistical significance for adjusted OR (*p* < 0.05). Abbreviations: OD, olfactory dysfunction; TD, taste disorder; NA, not applicable (missing data).

## Data Availability

Data are available on reasonable request.

## Supplementary Materials

The following supporting information can be downloaded at: https://www.mdpi.com/article/10.3390/life14030293/s1.
