# Supplementary material for: The Prognostic Value of Olfactory Dysfunction in Patients with COVID-19: The COVIDORA Study
Source: Life (Basel). 2024 Feb 22;14(3):293. doi: 10.3390/life14030293 (PMC10971396; doi:10.3390/life14030293)
Supplement: Supplementary file 1 [file life-14-00293-s001.zip › life-2872571-supplementary.pdf]

THE COVIDORA STUDY

CRF

INFORMATION FOR THE PATIENT

We will ASK YOU QUESTIONS in order to gather information about

- your medical history

- your recent symptoms

- about the quality of your ABILITY TO SMELL AND YOUR TASTE.

Answer freely based on your current condition.

Take time to think about each question before you give your answer. If you don't understand a question, ask us to explain it to you.

IDENTIFICATION PATIENT

First letter of the last name : __

First letter of the first name: __

Date of birth: |__|__| (day) |__|__| (month) |__|__|__|__| (year)

Age of the patient on the day of inclusion: |__|__| (years)

Sex: ☐ Female or ☐ Male

Date of inclusion: |__|__| (day) |__|__| (month) |__|__|__|__| (year)

Name of the investigator:

FIRST VISIT
Teleconsultation or face-to-face consultation
(to be carried out within 3 days of a positive COVID-19 diagnosis)

Date of the consultation: |__|__| (day) |__|__| (month) |__|__|__|__| (year)

CENTRE for inclusion = Centre number |__|__| (see the list below)?

CENTRE 1= Service ORL Lariboisière (Dr Hautefort),
CENTRE 2= Service ORL Henri Mondor (Dr Bartier et Dr Bequignon)
CENTRE 3= Service ORL Kremlin Bicêtre (Pr Papon et Dr Nevoux)
CENTRE 4= Service ORL La Pitié Salpetrière (Pr Nguyen)
CENTRE 5= Service Maladies Infectieuses, Hôtel Dieu (Pr Salmon)
CENTRE 6= Service ORL Centre Hospitalier Intercommunal de créteil (Pr Prulières Escabasse)
CENTRE 7= Hôpital Fondation Rotschild (Dr Corré)
CENTRE 8 =Service ORL, CHU de Strasbourg (Pr Debry)
CENTRE 9 = Service ORL Hospices civiles de Lyon (Pr Tringali)
CENTRE 10= Service ORL Hôpital Edouard Herriot-HCL Lyon (Pr Truy)
CENTRE 11= Service ORL Chu de Caen (Dr Patron)
CENTRE 12= Service ORL CHU Toulouse (Pr Serrano et Dr Debonnecaze)
CENTRE 13=Service ORL Institut Arthur Vernes, Paris (Dr Lecanu)
CENTRE 14= Service ORL CHU Nantes (Pr Malard)
CENTRE 15= Service d'ORL, CHU de La Conception, Marseille (Pr Michel)
CENTRE 16= Institut Universitaire de la Face et du Cou, Nice (Dr Vandersteen)
CENTRE 17= Service ORL Hôpital Saint-Philibert GHICL (Dr E Bartaire)
CENTRE 18 =Service ORL Hôpital Saint-Vincent de Paul GHICL / Faculté Libre de Médecine de Lille (Dr Deraedt)
CENTRE 19= Service ORL et chirurgie cervico-faciale CHU de Clermont-Ferrand (Dr Saroul)
CENTRE 20 = Service ORL, CHRU Nancy (Dr Rumeau)
CENTRE 21= Service ORL, CHU de Poitiers (Pr Dufour et Dr Carsuzaa)
CENTRE 22= Service ORL, Hôpital FOCH, Suresnes (Pr Hans)
CENTRE 23= Service ORL, CHU de Bordeaux, (Pr De Gabory)
CENTRE 24= Service ORL, Cochin (Dr Lacroix)

ACTUAL SITUATION

First of all, where is the patient on the date of Visit 1?
At home 0 At home with oxygen 1 Medical unit 2 ICU 3 Deceased 4

- At home ☐
- At home with oxygen☐
- Hospitalized in Medical unit with oxygen ☐
- Hospitalized in ICU ☐
- The patient is deceased ☐

If death, under what circumstances? ……………….

CARACTERISTIQUES EPIDEMIOLOGIQUES

Weight and Height

Weight |__|__|__| kg Height |__|,|__|__| m MBI |__|__|,|__| kg/m2

Profession

……………………………………………………………………

Tabacco

Do you smoke or have you ever been a smoker ? ☐ yes ☐ no
Current : 0 no 1 yes Consumption : |__|__|__|
weaned : 0 no 1 yes Number of years of weaning: |__|__|__|

General history

0 non 1 oui

- Allergies
  - **Do you have any known allergies?** ☐ yes ☐ no
  - If the answer is yes, which ones: ……………………………………………………

- Chronic illness
  - **Do you have a history of immunosuppression?** ☐ yes ☐ no
  - **Do you have a history of chronic illness?** ☐ yes ☐ no
  - If the answer is yes, which ones**:**
    ☐ Diabetes ☐ Hypertension ☐ Neoplasia: …………………………
    ☐ Renal insufficiency ☐ Inflammatory or degenerative neurological pathology
  - **Do you have a history of autoimmune disease (lupus, polyarthritis) ?** ☐ yes ☐ no

ENT history

Do you have an ENT history? ☐ yes ☐ no

- <u>Rhinological history</u>
  - If the answer is yes, which ones:

    ☐ **Allergic rhinitis:** 0 no 1 proven 2 suspected 3 NA
    If the answer is yes, which allergen is involved (mite, pollen?) ....
    If allergic to pollens : do you think you have started your seasonal allergy attack (appearance of your usual symptoms?) 0 no 1 yes
    ☐ **Chronic rhinosinusitis without polyps:** 0 no 1 yes
    ☐ **Chronic rhinosinusitis with polyps** 0 no 1 yes
    ☐ **Radiotherapy covering the head area :** 0 no 1 yes
    ☐ **Chemotherapy :** 0 no 1 yes

- <u>History of anosmia (complete loss of smell))</u>

  Do you have a history of complete loss of smell (anosmia) ? 0 no 1 yes
  If the answer is yes, are the symptoms old and permanent (older than 3 months)? 0 no 1 yes

- <u>History of head trauma</u>

  Do you have a history of severe head trauma?
  - 0 no 1 yes

Medication

0 non 1 oui

Are you taking any medication (including nasal spray) ? 0 no 1 yes
If the answer is yes, which ones: ………………………………………………………………………

CLINICAL FEATURES

Date of onset of first COVID symptoms

When did you first experience symptoms attributable to COVID-19?____/ ____ / ____

Olfaction disorders

Do you have a sense of smell disorder. (Do you have difficulty smelling odors?). 0 no 1 yes

**If the answer is yes, when did it start?**	____/ ____ / ____
<u>Onset of the symptom</u><br>0 gradually 1 suddenly<br>**Did the smell problems appear gradually or suddenly?**	☐ Gradually<br>☐ Suddenly

Inaugural nature of the symptom<br>0 no 1 yes<br>**Were the problems with your sense of smell the first of your symptoms in isolation?**<br>**If the answer is no, has it been:**<br>**- Concomitant with other symptoms?**<br>**- Preceded by classical symptoms of COVID-19 (fever >38°, cough?)**<br>**- Preceded by minor symptoms (fatigue, aches, headaches?)**	☐ Yes ☐ No<br>☐ Yes ☐ No<br>☐ Yes ☐ No<br>☐ Yes ☐ No
Quantitative dysosmia<br>1 partial (= hyposmia)<br>2 total (= anosmia)<br>**Is your loss of smell partial or total?**	☐ Partial ☐ Total
Parosmia<br>0 no 1 yes<br>**Did you ever perceive a smell that did not correspond to the olfactory stimulation? (For example, coffee smells like burnt rubber)**	☐ Yes ☐ No
Phantosmia<br>0 no 1 yes<br>**Have you ever perceived a smell without any olfactory stimulation?**	☐ Yes ☐ No
**Can you accurately smell the scent of:**<br>0 no 1 yes<br>• **Cinnamon ?**<br>• **Cumin ?**<br>• **Cloves ?**<br>• **Vanilla ?**<br>• **Vinegar ?**	☐ Yes ☐ No<br>☐ Yes ☐ No<br>☐ Yes ☐ No<br>☐ Yes ☐ No<br>☐ Yes ☐ No

Evolution of the olfaction disorder

How long has your loss of smell been evolving in days ?..

Rhinological symptoms

Rhinorrhea<br>If yes, concomitant to the smell disorder?<br>0 no 1 yes<br>**Did you have a runny nose?**<br>0 no 1 yes<br>**If so, was it concomitant with the loss of smell?**	☐ Yes ☐ No<br>☐ Yes ☐ No<br>☐ Yes ☐ No<br>☐ Yes ☐ No
Nasal obstruction<br>0 no 1 yes<br>**Did you have the feeling that your nose was completely blocked?**	☐ Yes ☐ No
Nasal hyperreactivity<br>0 no 1 yes<br>**Did you have an itchy nose with the urge to sneeze +/- sneezing?**	☐ Yes ☐ No

Nasal paresthesia (burning sensation, intranasal pain)<br>**Did you experience a sensation of burning nose, tingling, pain?**	☐ Yes ☐ No
Other symptoms<br>0 no 1 yes<br>**Did you experience any other rhinologic symptoms?**<br>If the answer is yes, which ones:	☐ Yes ☐ No<br>…………………………<br>………………………………

Taste disorder

Dysgueusia<br>0 no 1 yes<br>**Did you experience a taste disorder?**<br>**If the answer is yes:**<br>- **Was it about the flavors of the food?**<br>- **Was it about salty/sweet/bitter/sour?**	☐ Yes ☐ No<br><br>☐ Yes ☐ No<br>☐ Yes ☐ No
**Can you recognize:**<br>0 no 1 yes<br>• **Salty taste ?**<br>• **Sweet taste ?**<br>• **Bitter taste ?**<br>• **Acidic taste ?**	 <br>☐ Yes ☐ No<br>☐ Yes ☐ No<br>☐ Yes ☐ No<br>☐ Yes ☐ No

Other symptoms of COVID-19

Did you experience: ….? 0 no 1 yes

• **Fever**	☐ Yes ☐ No	• **Difficulty breathing (dyspnea?)**	☐ Yes ☐ No
• **Coughing**	☐ Yes ☐ No	• **Diarrhea**	☐ Yes ☐ No
• **Headache**	☐ Yes ☐ No	• **Nausea/vomiting**	☐ Yes ☐ No
• **Fatigue**	☐ Yes ☐ No	• **Dizziness**	☐ Yes ☐ No
• **Muscle pain**	☐ Yes ☐ No	• **Chest pain/oppression**	☐ Yes ☐ No
• **Sore throat**	☐ Yes ☐ No	• **Joint pain**	☐ Yes ☐ No
• **Conjunctivitis**	☐ Yes ☐ No	• **Skin rash**	☐ Yes ☐ No
• **Laryngitis**	☐ Yes ☐ No	• **Other...**<br>**If the answer is yes, which one :**	☐ Yes ☐ No<br>………………

Diagnosis of COVID-19

Contagion
0 no 1 yes
Have you been in contact with a known case of COVID-19? ☐ Yes ☐ No

Screening
0 no 1 yes unless otherwise specified

Swab<br>• **Have you been swabbed for COVID-19?**<br>**If the answer is yes :**<br>**- Nasal or pharyngeal swab (in the throat)**<br>**- On what date?**<br>**- What was the test result? 0 negative 1 positive**<br>**Have you been swabbed a second time for COVID-19?**<br>**If the answer is yes :**<br>**- Nasal or pharyngeal swab (in the throat)**<br>**- On what date?**<br>**- What was the test result? 0 negative 1 positive**	☐ Yes ☐ No<br><br>☐ Nasal ☐ Throat<br>____/ ____ / ____<br>☐ Negative ☐ Positive<br>☐ Yes ☐ No<br><br>☐ Nasal ☐ Throat<br>____/ ____ / ____<br>☐ Negative ☐ Positive
Imaging<br>• **Did you have a chest CT-scan?**<br>**- If so, on what date?**<br>**- What was the result?**<br>**- If positive, what were the signs suggestive of COVID?**	 <br>☐ Yes ☐ No<br>☐ Negative ☐ Positive<br>...................................

SECOND VISIT
Teleconsultation or face-to-face consultation
(to be carried out between days 7 and 10 of a positive COVID-19 diagnosis)

Date of the consultation: |__|__| (jour) |__|__| (mois) |__|__|__|__| (année)

ACTUAL SITUATION

First of all, where is the patient on the date of Visit 2 ?
At home 0 At home with oxygene 1 Medical unit 2 ICU 3 Deceased 4
- At home ☐
- At home with oxygen☐
- Hospitalized in Medical unit with oxygene ☐
- Hospitalized in ICU ☐
- The patient is deceased ☐

If death, under what circumstances? ………………

Management evolution

0 no 1 yes unless otherwise specified

• **Have you been hospitalized?**<br>**If yes, in which type of medical unit?**	☐ Yes ☐ No<br>……………………………………

Evolution of other symptoms of COVID-19

Since first visit, did you experience : ? 0 non 1 oui

• **Fever**	☐ Yes ☐ No	• **Difficulty breathing (dyspnea?)**	☐ Yes ☐ No
• **Coughing**	☐ Yes ☐ No	• **Diarrhea**	☐ Yes ☐ No
• **Headache**	☐ Yes ☐ No	• **Nausea/vomiting**	☐ Yes ☐ No
• **Fatigue**	☐ Yes ☐ No	• **Dizziness**	☐ Yes ☐ No
• **Muscle pain**	☐ Yes ☐ No	• **Chest pain/oppression**	☐ Yes ☐ No
• **Sore throat**	☐ Yes ☐ No	• **Joint pain**	☐ Yes ☐ No
• **Conjunctivitis**	☐ Yes ☐ No	• **Skin rash**	☐ Yes ☐ No
• **Laryngitis**	☐ Yes ☐ No	• **Other…**	☐ Yes ☐ No
		**If the answer is yes, which one** :	………………

Si pas de perte d'odorat dépistée à la 1ère visite, reposez les questions suivantes :

Since the last consultation, have you noticed the appearance of a problem with your sense of smell (do you have difficulty smelling odors?) ☐ Yes ☐ No

Olfaction disorders

**If the answer is yes, when did it start?**	____/ ____ / ____
Onset of the symptom<br>0 gradually 1 suddenly<br>**Did the smell problems appear gradually or suddenly?**	☐ Gradually<br>☐ Suddenly
Inaugural nature of the symptom<br>0 no 1 yes<br>**Were the problems with your sense of smell the first of your symptoms in isolation?**<br>**If the answer is no, has it been:**<br>- **Concomitant with other symptoms?**<br>- **Preceded by classical symptoms of COVID-19 (fever >38°, cough?)**<br>- **Preceded by minor symptoms (fatigue, aches, headaches?)**	☐ Yes ☐ No<br><br><br>☐ Yes ☐ No<br>☐ Yes ☐ No<br>☐ Yes ☐ No
Quantitative dysosmia<br>1 partial (= hyposmia) 2 total (= anosmia)<br>**Is your loss of smell partial or total?**	☐ Partial ☐ Total
Parosmia<br>0 no 1 yes<br>**Did you ever perceive a smell that did not correspond to the olfactory stimulation? (For example, coffee smells like burnt rubber)**	☐ Yes ☐ No

Phantosmia<br>0 no 1 yes<br>**Have you ever perceived a smell without any olfactory stimulation?**	☐ Yes ☐ No
**Can you accurately smell the scent of :**<br>0 no 1 yes<br>• **Cinnamon ?**<br>• **Cumin ?**<br>• **Cloves ?**<br>• **Vanilla ?**<br>• **Vinegar ?**	☐ Yes ☐ No<br>☐ Yes ☐ No<br>☐ Yes ☐ No<br>☐ Yes ☐ No<br>☐ Yes ☐ No

Evolution of the olfaction disorder

How long has your loss of smell been evolving in days ?………………………………………

Rhinological symptoms

Rhinorrhea<br>If yes, concomitant to the smell disorder?<br>0 no 1 yes<br>**Did you have a runny nose?**<br>0 no 1 yes<br>**If so, was it concomitant with the loss of smell?**	☐ Yes ☐ No<br>☐ Yes ☐ No<br>☐ Yes ☐ No<br>☐ Yes ☐ No
Nasal obstruction<br>0 no 1 yes<br>**Did you have the feeling that your nose was completely blocked?**	☐ Yes ☐ No
Nasal hyperreactivity<br>0 no 1 yes<br>**Did you have an itchy nose with the urge to sneeze +/- sneezing?**	☐ Yes ☐ No
Nasal paresthesia (burning sensation, intranasal pain)<br>**Did you experience a sensation of burning nose, tingling, pain?**	☐ Yes ☐ No
Other symptoms<br>0 no 1 yes<br>**Did you experience any other rhinologic symptoms?**<br>If the answer is yes, which ones:	☐ Yes ☐ No<br>…………………………

Taste disorder

Dysgueusia<br>0 no 1 yes<br>**Did you experience a taste disorder?**<br>**If the answer is yes:**<br>- **Was it about the flavors of the food?**<br>- **Was it about salty/sweet/bitter/sour?**	☐ Yes ☐ No<br>☐ Yes ☐ No<br>☐ Yes ☐ No

**Can you recognize:**<br>0 no 1 yes	
• **Salty taste ?**	☐ Yes ☐ No
• **Sweet taste ?**	☐ Yes ☐ No
• **Bitter taste ?**	☐ Yes ☐ No
• **Acidic taste ?**	☐ Yes ☐ No

Other symptoms of COVID-19

Did you experience: ….? 0 no 1 yes

• **Fever**	☐ Yes ☐ No	• **Difficulty breathing (dyspnea?)**	☐ Yes ☐ No
• **Coughing**	☐ Yes ☐ No	• **Diarrhea**	☐ Yes ☐ No
• **Headache**	☐ Yes ☐ No	• **Nausea/vomiting**	☐ Yes ☐ No
• **Fatigue**	☐ Yes ☐ No	• **Dizziness**	☐ Yes ☐ No
• **Muscle pain**	☐ Yes ☐ No	• **Chest pain/oppression**	☐ Yes ☐ No
• **Sore throat**	☐ Yes ☐ No	• **Joint pain**	☐ Yes ☐ No
• **Conjunctivitis**	☐ Yes ☐ No	• **Skin rash**	☐ Yes ☐ No
• **Laryngitis**	☐ Yes ☐ No	• **Other...**	☐ Yes ☐ No
		**If the answer is yes, which one :**	………………

If loss of smell is detected at the first visit, ask the following questions:

Treatment received for loss of smell / Rehabilitation

Have you received treatment for your loss of smell ? ☐ Yes ☐ No

If yes, which ones ? 0 non 1 oui

	Oui / Non	**Si oui lequel ?**	**Si oui durée ?**
**Treatment with systemic corticosteroid therapy (oral) ?**	☐ Yes ☐ No	….	……
**Local treatment ?**			…….
• Nose washes with saline solution	☐ Yes ☐ No	….	
• Local corticosteroid therapy	☐ Yes ☐ No	….	
• Other treatments ?	☐ Yes ☐ No		
**Anti-COVID treatment (plaquenil, zithromax , autre antiviral)**	☐ Yes ☐ No	….	…….

- **Have you done olfactory rehabilitation?** ☐ Yes ☐ No
- **If yes, for how long in days : ……………..**

Evaluation of olfactory recovery if disorder already described initially

- **How far are we from the beginning of the disorder of the sense of smell (in days) ?**: …….

- **To date, have you recovered in terms of olfaction?** ☐ Yes ☐ No
  If so, in your opinion, have you recovered partially or totally? ☐ Partially ☐ Totally
  0 partially 1 totally
  If so, how would you rate your recovery on a scale of 0 to 10
  (10= complete recovery) ?...................

- **Have you ever perceived a smell that did not correspond to the olfactory stimulation (parosmia)?**
  ☐ Yes ☐ No

- **Have you ever perceived a smell without any olfactory stimulation (phantosmia)?**
  ☐ Yes ☐ No

- **How long did it take for your smell to recover in days?**

[0-5[days	[5-10[days	[10-15[days	[15-30[days	More than a month
